# A nomogram based on pretreatment CT radiomics features for predicting complete response to chemoradiotherapy in patients with esophageal squamous cell cancer

**DOI:** 10.1186/s13014-020-01692-3

**Published:** 2020-10-29

**Authors:** He-San Luo, Shao-Fu Huang, Hong-Yao Xu, Xu-Yuan Li, Sheng-Xi Wu, De-Hua Wu

**Affiliations:** 1 Department of Radiation Oncology, Nanfang Hospital, Southern Medical University, Guangzhou, 510515 Guangdong Province China; 2 Department of Radiotherapy, Shantou Central Hospital, Shantou, Guangdong China; 3 Department of Medical Oncology, Shantou Central Hospital, Shantou, Guangdong China

**Keywords:** Chemo-radiotherapy, Esophageal squamous cell cancer, Radiomics, Response, Nomogram

## Abstract

**Purpose:**

To develop and validate a nomogram model to predict complete response (CR) after concurrent chemoradiotherapy (CCRT) in esophageal squamous cell carcinoma (ESCC) patients using pretreatment CT radiomic features.

**Methods:**

Data of patients diagnosed as ESCC and treated with CCRT in Shantou Central Hospital during the period from January 2013 to December 2015 were retrospectively collected. Eligible patients were included in this study and randomize divided into a training set and a validation set after successive screening. The least absolute shrinkage and selection operator (LASSO) with logistic regression to select radiomics features calculating Rad-score in the training set. The logistic regression analysis was performed to identify the predictive clinical factors for developing a nomogram model. The area under the receiver operating characteristic curves (AUC) was used to assess the performance of the predictive nomogram model and decision curve was used to analyze the impact of the nomogram model on clinical treatment decisions.

**Results:**

A total of 226 patients were included and randomly divided into two groups, 160 patients in training set and 66 patients in validation set. After LASSO analysis, seven radiomics features were screened out to develop a radiomics signature Rad-score. The AUC of Rad-score was 0.812 (95% CI 0.742–0.869, *p* < 0.001) in the training set and 0.744 (95% CI 0.632–0.851, *p* = 0.003) in the validation set. Multivariate analysis showed that Rad-score and clinical staging were independent predictors of CR status, with *p* values of 0.035 and 0.023, respectively. A nomogram model incorporating Rad-socre and clinical staging was developed and validated, with an AUC of 0.844 (95% CI 0.779–0.897) in the training set and 0.807 (95% CI 0.691–0.894) in the validation set. Delong test showed that the nomogram model was significantly superior to the clinical staging, with *p* < 0.001 in the training set and *p* = 0.026 in the validation set. The decision curve showed that the nomogram model was superior to the clinical staging when the risk threshold was greater than 25%.

**Conclusion:**

We developed and validated a nomogram model for predicting CR status of ESCC patients after CCRT. The nomogram model was combined radiomics signature Rad-score and clinical staging. This model provided us with an economical and simple method for evaluating the response of chemoradiotherapy for patients with ESCC.

## Introduction

Esophageal cancer (EC) is one of the most common digestive malignant tumors, ranking seventh in terms of incidence and sixth in mortality overall [1]. Esophagostomy is the mainstay of treatment option for early esophageal cancer [2]. Unfortunately, most patients diagnosed as locally advanced esophageal cancers lost the opportunity for surgery at the time of diagnosis, for which concurrent chemo-radiotherapy (CCRT) has been recommended as a standard treatment [3]. However, the effect of CCRT remained poor, as more than a half of patients treated with standard-dose CCRT were eventually developed recurrence or distant metastases and succumbed to this disease [4, 5]. On the other hand, patients who achieved clinical complete response (CR) may obtain long-term survival [6, 7]. Therefore, early identification of patients who would achieve CR and who are at risk of poor response before CCRT would allow personalization of their treatment.

For patients received CCRT, clinical stage is the most important factor for prognosis [8]. Recently, a series of clinical biomarkers have been explored and validated to be used in prediction of therapeutic response [9–11]. Radiomics is an image analysis technology that extracts quantitative features from computed tomography (CT) images, magnetic resonance (MR) images, positron emission tomography (PET) images, etc. [12]. Several studies have demonstrated that radiomics could be applied in prediction of treatment response for patients with EC. Yip et al. [13] combined CT-based texture feature and esophageal maximal wall thickness assessment to predict the overall survival in a cohort of 31 EC patients treated with CCRT. As a result, the model performed better than treatment response alone. Jin et al. [14] developed and validated a model combined radiomic features with dosimetric parameters to predict the treatment response of patients with EC who underwent CCRT with promising results.

However, most radiomic studies enrolled a small number of investigated patients and included both adenocarcinoma and squamous cell carcinoma patients. As we know, these two histological types of EC may present different sensitive to CCRT. In this study, we used quantitative radiomics features based on pretreatment CT to develop a model to predict CR to CCRT in patients with esophageal squamous cell carcinoma (ESCC). This model may help doctors to make the best therapeutic management.

## Patients and methods

### Patients’ selection and randomization

Data of patients diagnosed as esophageal squamous cell carcinoma (ESCC) and treated with definitive chemoradiotherapy (dCRT) in Shantou Central Hospital during the period from January 2013 to December 2015 were retrospectively collected. Eligible patients were included in this study after successive screening, and the patients were excluded if they met the exclusion criteria as followed: (1) patients with distant metastatic disease; (2) patients received low-dose palliative radiotherapy; (3) patients received preoperative or postoperative adjuvant radiotherapy; (4) patients had incomplete clinicopathological information; (5) patients diagnosed as esophageal fistula and received esophageal stent implantation; (6) poor visualization quality due to image artifacts or the tumor was too small to be recognized on CT images; (7) patients had other primary tumor.

**Fig. 1:**
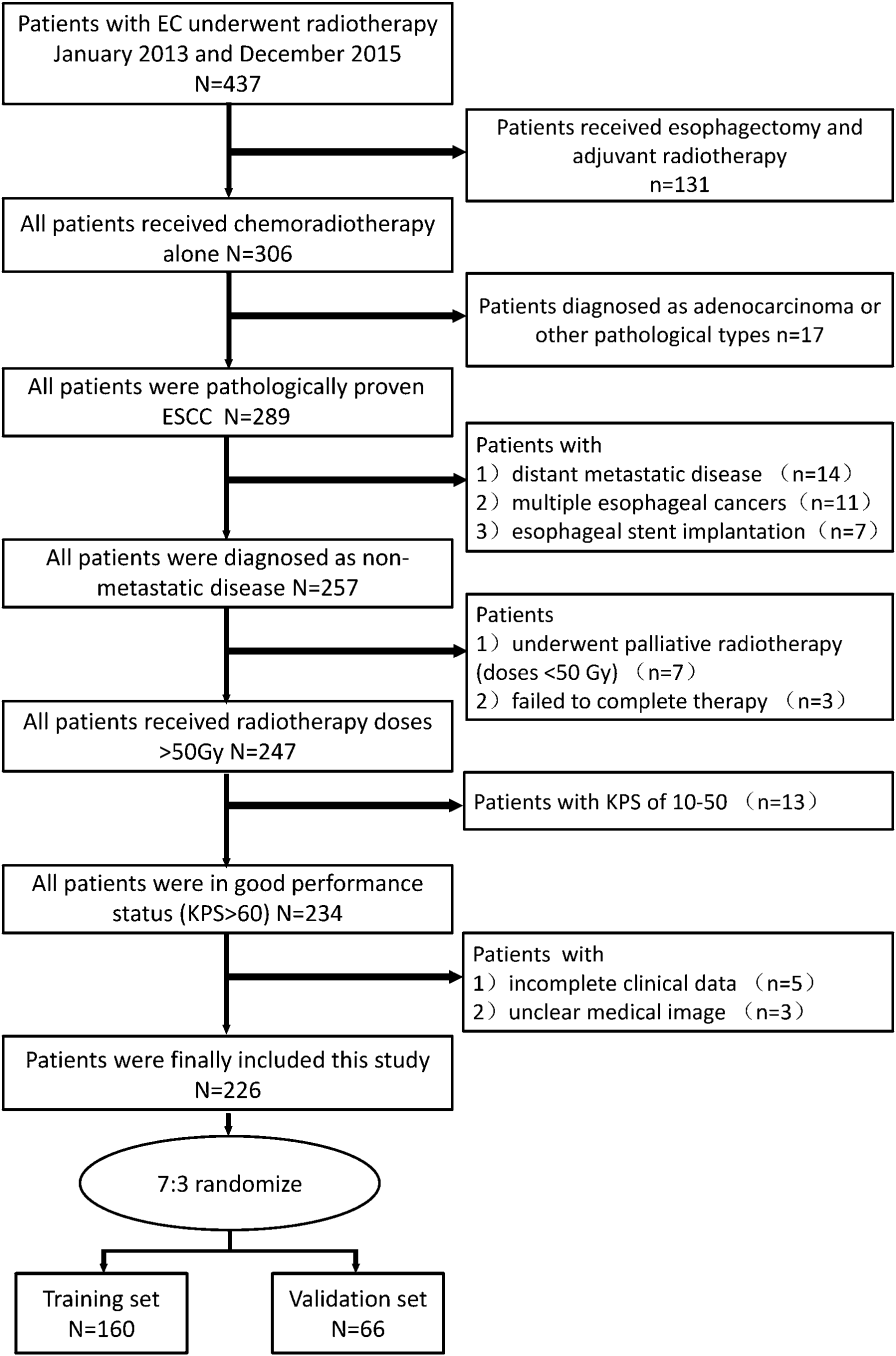
Flow chart of patients’ selection and randomization

A total of 226 patients were included in the final analysis, and the enrolled patients were randomly divided into two groups, with 160 patients in the training set and 66 patients in the validation set. To maximize the generalization ability of the model, stratification was used to keep the class proportions intact according to various variables. The process of patients’ selection and randomization were shown in Fig. 1. This study was approved by the Institutional Committee of the Shantou Central Hospital on Human Rights. Disease of the patients was staged according to the 8th edition of AJCC TNM classification for esophageal cancer [15].

### Chemoradiotherapy

All patients were treated with three-dimensional conformal radiation therapy (3DRT) or intensity-modulated radiation therapy (IMRT) technique in this study. A Varian IX or Varian 23EX linear accelerator was used to deliver the radiotherapy treatment plan. The gross tumor volume (GTV) includes the esophageal cancer (GTVp) and the positive regional lymph nodes (GTVnd). The GTV was determined by CT, barium esophagogram, endoscopic examination or PET imaging. The CTV is defined as GTVp with a 0.5–1 cm radial margin and a 2.5–3 cm proximal and distal margin and the GTVnd with a 0.5–0.8 cm margin. The planning target volume (PTV) was determined by adding a 0.5–1 cm margin to CTV. A total prescribed dose of 50–72 Gy (median, 64 Gy) in 25–36 fractions was delivered in 5 fractions per week.

Two cycles of platinum-based chemotherapy combined with 5-fluorouracil or a taxane (docetaxel or paclitaxel) were administered on the patients concurrently with radiotherapy. Sixty one patients received TP (paclitaxel + cisplatin) chemotherapy, which consists of cisplatin (60 mg/m^2^ on Day 1) plus paclitaxel (135–180 mg/m^2^ on Days 1). One hundred and sixty five patients received the PF (cisplatin + fluorouracil) regimen, which consists of cisplatin (60 mg/m^2^ on Day 1) and fluorouracil (750 mg/m^2^/24 h on Days 1–4).

### Response assessment

The response of these patients to treatment was evaluated one month after CRT according to the criteria of short-term radiotherapeutic effect evaluation standard on esophageal cancer by CT images and barium esophagogram. According to the assessment criteria, clinical response was classified as complete response (CR), partial response (PR), no response (NR), or progressive disease (PD). Patients who had a CR as evaluated by barium esophagogram and had the maximal esophageal wall thickness of ≤ 1.2 cm and the volumes of residual lymph nodes of ≤ 1.0 cm^3^ on CT were defined as CR [16].

### CT image radiomic feature extraction

CT image acquisitions were performed before radiotherapy. All patients were scanned using GE Lightspeed 64-slice spiral CT (GE Medical systems, Milwaukee, Wis) according to the following acquisition parameters: The CT tube voltage was 120 kV and the tube current was 120 mAs. Rack rotation time: 0.6 s; Detector collimation parameters: 64 × 0.625 mm; field of view (FOV): 400–500 mm; Matrix: 512 × 512; Layer thickness is 5 mm, layer spacing is 5 mm. Contrast medium was injected with a high pressure syringe at a flow rate of 3.0 ml/s (1–1.5 ml/kg, ioproxamine injection 300), followed by 30–40 ml of normal saline for flushing, and late arterial CT images were collected with a delay of 30 s. To minimize the variability among different images, scan were resampled to voxel of 1 × 1 × 1 mm^3^.

To obtain volume of interest (VOIs) for further radiomic analysis, a 3DSlicer (version, 4.10.2, Stable Release) with its extension (radiomics) was used for image segmentation. The contours of VOI were consistent with gross tumor volume (GTV) delineated by radiation oncologists for radiotherapy treatment planning design. Any pixel with an attenuation of less than − 50 HU was excluded to avoid adjacent air, fat, blood vessels and surrounding organs. In order to assess their robustness of radiomics feature, image segmentation was performed independently by a radiation oncologist and another radiologist. To evaluate the reproducibility of the radiomics analysis, tumor segmentation was repeated two months later by the same observer for 30 randomly chosen patients.

Pyradiomics V3.6.2 was used to extract radiomic features from delineated VOIs. Using this package, several categories features were extracted from VOIs, including first order statistics features (IH, intensity histogram), shape-based histogram features, and texture features (gray-level co-occurrence matrix, GLCM; gray-level size-zone matrix, GLSZM; gray-level run-length matrix, GLRLM; neighboring gray-tone difference matrix, NGTDM; and gray-level dependence matrix, GLDM). The wavelet filter was used in image pre-processing and the texture features were extracted from the images preprocessed. In all, for each VOI, 107 original features (Additional file 1: Supplemental Table 1) and 744 wavelet features (Additional file 1: Supplemental Table 1) were collected. Among 107 original features, there were 18 first order statistics features, 14 shape-based histogram features, 24 GLCM features, 14 GLDM features, 16 GLRLM features、16 GLSZM features and 5 NGTDM features. Mathematical definitions of these radiomic features have previously been described [17] and available at https://pyradiomics.readthedocs.io/en/latest/features.html.

### Statistical analysis

Statistical analyses were performed using R software version 3.6.2 (R Foundation for Statistical Computing, Vienna, Austria). The difference in the clinical characteristics between training set and validation set was determined by Chi-squared test or Fisher’s. A *p* value of < 0.05 was considered statistically significant.

After features extraction, all the radiomic features were normalized using Z-score normalization, by which the feature values were centered by removing the mean value of each feature, then divided by the standard deviation of each features. The pre-processing made feature values lie within similar ranges, which reduced the influence of features with large discrete values. The intra-class correlation coefficient (ICC) analysis was performed to evaluate the reproducibility of each radiomics feature. Only the features with ICCs value ≥ 0.900 were selected for further analysis. Then, the least absolute shrinkage and selection operator (LASSO) with logistic regression was applied to identify the optimal features to predict CR in the training set patients. The hyper parameter lambda (λ) was chosen based on tenfold cross-validation with the smallest mean squared error. We calculated a radiomics feature score (Rad-score) for each patient based on the coefficients weighted by LASSO logistic regression model in the training set. The LASSO logistic regression formula:

$${\text{Rad-score}} =\upbeta 0 +\upbeta {\text{1X1}} +\upbeta {\text{2X2}} +\upbeta {\text{3X3}} + \cdots +\upbeta {\text{nXn}}.$$

In the above formula, Xn represents the radiomics feature identified by the LASSO-Logistic regression model, β0 is the constant for Rad-score, and βn is the regression coefficient of the corresponding feature in the regression model. The Rad-score for each patient can be calculated according to the formula. Receiver operating characteristic (ROC) curve analysis was used to assess the performance of Rad-core.

Univariate analysis was performed using correlation analysis. The Mann–Whitney U test was used to evaluate potential relationships between the clinical factors and CR status in the training and validation set. Multivariable logistic regression analysis was performed to screen out the predictor for CR. A nomogram model predicting CR was developed based on the multivariable logistic regression analysis using rms package and foreign package in R software. The model performance was assessed by the ROC curve analysis using pROC package in R software in both the training and testing groups. Comparison of ROCs was performed using Delong test. Decision curve analysis was conducted to determine the clinical usefulness of the radiomics-based nomogram and clinical stage by quantifying the net benefits at different threshold probabilities in the validation dataset.

## Results

### Patients’ characteristics

Patients’ characteristics are listed in Table 1. A total of 226 ESCC patients who received chemoradiotherapy in our hospital met the inclusion criteria and were included in this study. They were randomly divided into training set and validation set. There are 160 patients enrolled in the training set and 66 patients in the validation set. There were no significant differences in the distribution of baseline characteristics such as age, gender, tumor location, T stage, N stage, clinical staging, lactate dehydrogenase (LDH), neutrophil to 1ymphocyte ratio (NLR) and platelet to lymphocyte ratio (PLR). The results were not statistically significant (*p* > 0.05), and the two groups of patients were comparable. In addition, the ratio of patients who achieved clinical CR after chemo-radiotherapy was 35.0% in the training set and 33.3% in the validation set, and the difference was not statistically significant (*p* = 0.624).

**Table 1:** Comparison of patients’ characteristics between training set and test set

Variables	Training set (n = 160)	Validation set (n = 66)	χ^2^/*t*	*P*
Age (years), mean ± SD	65.12 ± 10.22	66.20 ± 9.23	− 0.741	0.459
Gender			0.004	0.951
Male	117 (73.1)	48 (72.7)		
Female	43 (26.9)	18 (27.3)		
Tumor location			1.120	0.772
Cervical	10 (6.3)	4 (6.1)		
Upper thoracic	39 (24.4)	12 (18.2)		
Middle thoracic	87 (54.4)	40 (60.6)		
Lower thoracic	24 (15.0)	10 (15.2)		
T stage			1.362	0.715
T1	1 (0.6)	1 (1.5)		
T2	16 (10.0)	4 (6.1)		
T3	65 (40.6)	29 (43.9)		
T4	78 (48.8)	32 (48.5)		
N stage			0.522	0.914
N0	23 (14.4)	11 (16.7)		
N1	71 (44.4)	27 (40.9)		
N2	54 (33.8)	24 (36.4)		
N3	12 (7.5)	4 (6.1)		
Clinical stage			1.759	0.624
I	1 (0.6)	1 (1.5)		
II	17 (10.6)	10 (15.2)		
III	92 (57.5)	33 (50.0)		
Iva	50 (31.3)	22 (33.3)		
Radiation dose, median (range)	64 (60–66)	64 (60–66)	− 0.630	0.529
Chemotherapy regimen			1.580	0.209
PF	113 (68.5)	52 (31.5)		
TP	47 (77.0)	14 (23.0)		
LDH group			3.189	0.074
High	79 (50.6)	42 (63.6)		
Normal	81 (49.4)	24 (36.4)		
NLR, median (range)	2.73 (2.00–3.71)	2.82 (1.82–3.71)	− 0.149	0.882
PLR, median (range)	134.49 (102.43–176.55)	144.47 (96.72–196.38)	0.571	0.568
CR ratio	56 (35.0)	22 (33.3)	0.057	0.811
Rad-score, mean ± SD	− 16.1105 ± 4.03384	− 15.8565 ± 3.69877	− 0.441	0.660

### Rad-score building based on radiomics features

**Fig. 2:**
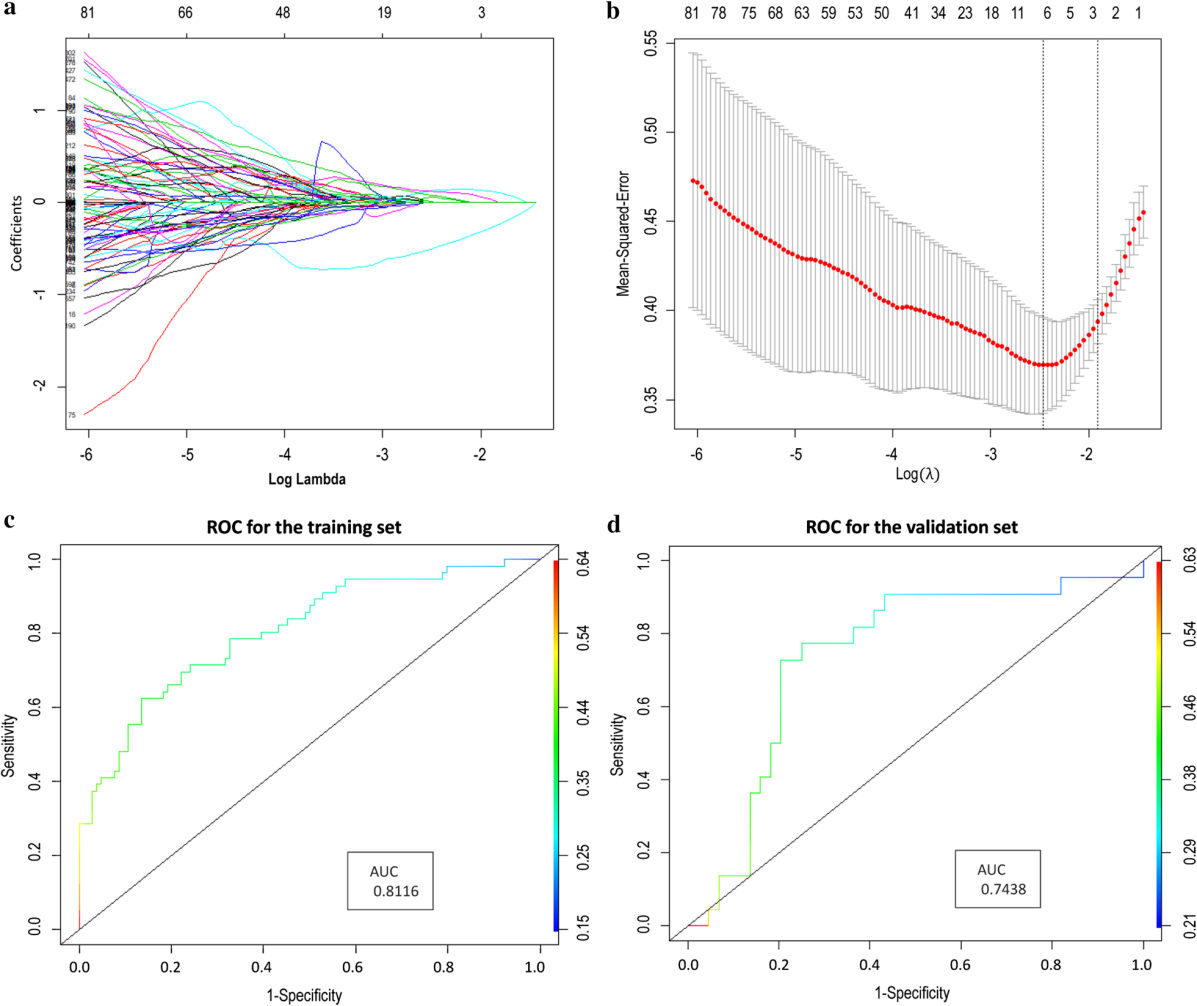
Selection of radiomics features for predicting CR using the LASSO logistic regression model. **a** LASSO coefficient profiles of the radiomicis features. **b** The cross validation curve. **c** ROC for Rad-score in training set. **d** ROC for Rad-score in validation set

LASSO-logistic regression was used to reduce the dimensionality of the extracted radiomics features and screen out the optimal radiomics features for predicting the patient's CR in the training set (Fig. 2a, b). As a result, seven radiomics features were selected (The features and their coefficients were listed in the Table 2). In order to ensure the reproducibility of the features, intra-observer ICCs and inter-observer ICCs was calculated. The inter-observers ICCs and intra-observers ICCs of seven features were listed in the Table 3. Using the filter criteria of ICCs > 0.900, the seven features were stable for further analysis (ICC range: 0.904–0.978). The Rad-score was calculated as follows: Rad-score = − 0.6786125363–0.4893651433*original_shape_MinorAxisLength + 0.0009304499* original_GLDM_DependenceNonUniformityNormalized + 0.1346597598* wavelet-LHH_NGTDM_Coarseness + 0.1197247331*wavelet-LHH_NGTDM_Contrast + 0.0018481705*wavelet-HLH_NGTDM_Coarseness + 0.0749703324*wavelet-HHL_GLDM_SmallDependenceLowGrayLevelEmphasis + 0.0557647640*wavelet-LLL_GLDM_DependenceNonUniformityNormalized.

**Table 2:** The radiomics features selected by LASSO regression analysis

Radiomics features	Coefficients
Original_shape_MinorAxisLength	0.4893651433
Original_GLDM_DependenceNonUniformityNormalized	0.0009304499
Wavelet-LHH_NGTDM_Coarseness	0.1346597598
Wavelet-LHH_NGTDM_Contrast	0.1197247331
Wavelet-HLH_NGTDM_Coarseness	0.0018481705
Wavelet-HHL_GLDM_SmallDependenceLowGrayLevelEmphasis	0.0749703324
Wavelet-LLL_GLDM_DependenceNonUniformityNormalized	0.0557647640

**Table 3:** Reproducibility of the radiomics features selected by LASSO regression analysis

Radiomics features	Reproducibility	
	Intra-observer-ICC (95% CI)	Inter-observer-ICC (95% CI)
Original_shape_MinorAxisLength	0.975 (0.949–0.988)	0.949 (0.896–0.975)
Original_GLDM_DependenceNonUniformityNormalized	0.940 (0.879–0.971)	0.913 (0.826–0.958)
Wavelet-LHH_NGTDM_Coarseness	0.978 (0.954–0.989)	0.954 (0.905–0.978)
Wavelet-LHH_NGTDM_Contrast	0.963 (0.924–0.982)	0.939 (0.876–0.971)
Wavelet-HLH_NGTDM_Coarseness	0.954 (0.907–0.978)	0.921 (0.841–0.962)
Wavelet-HHL_GLDM_SmallDependenceLowGrayLevelEmphasis	0.949 (0.897–0.976)	0.937 (0.872–0.969)
Wavelet-LLL_GLDM_DependenceNonUniformityNormalized	0.922 (0.843–0.962)	0.904 (0.809–0.953)

ROC curve analysis was used to evaluate the predictive performance of Rad-score for CR. As shown in Fig. 2c, d, the AUCs of the Rad-score predicted CR status in the training set and test set were 0.812 (95% CI 0.742–0.869, *p* < 0.001) and AUC = 0.744 (95% CI 0.632–0.851, *p* = 0.003), respectively.

### Development and validation of a predictive nomogram based on Rad-score

We conducted univariate and t multivariate analyses to identify predictive factors for CR status in training set. Univariate analysis showed that the clinical staging of the patients was significantly associated with CR status. However, CR status was not related with age, sex, tumor location, radiation dose, serum LDH level, NLR and PLR (As shown in Table 4, all *p* > 0.05). The results were validated in validation set. Therefore, the clinical staging and Rad-score were enrolled into the logistic multivariate analysis model. The results of multivariate analysis showed that the clinical staging and Rad-score were independent predictors of CR status for ESCC patients treated with CCRT both in training set and validation set (as shown in Table 5, all *p* < 0.05).

**Table 4:** The association between clinicopathological characteristics and CR status in ESCC patients received CCRT

Variables	Training set (n = 160)		*P*	Validation set (n = 66)		*P*
	CR	Non-CR		CR	Non-CR	
Age (years), mean ± SD	66.18 ± 9.55	64.55 ± 10.56	0.337	65.64 ± 10.09	66.48 ± 8.88	0.730
Gender			0.064			0.258
Male	36 (30.8)	81 (69.2)		15 (31.2)	33 (68.8)	
Female	20 (46.5)	23 (53.5)		7 (38.9)	11 (61.1)	
Tumor location			0.221			0.216
Cervical	4 (40.0)	6 (60.0)		2 (50)	2 (50)	
Upper thoracic	16 (41.0)	23 (59.0)		6 (50)	6 (50)	
Middle thoracic	32 (36.8)	55 (63.2)		13 (32.5)	27 (67.5)	
Lower thoracic	4 (16.7)	20 (83.3)		1 (50%)	1 (50%)	
Clinical stage			< 0.001			0.016
I	1 (100.0)	0 (0)		1 (100.0)	0 (0)	
II	12 (70.6)	5 (29.4)		5 (50.0)	5 (50.0)	
III	40 (43.5)	52 (56.5)		14 (42.4)	19 (57.6)	
Iva	3 (6.0)	47 (94.0)		2 (9.1)	44 (90.9)	
Radiation dose, median (range)	64 (61.25–66)	64 (62–66)	0.221	63 (60–65.75)	63 (60–65.75)	0.737
Chemotherapy regimen			0.209			0.831
PF	43 (38.1)	70 (61.9)		17 (32.7)	35 (67.3)	
TP	13 (27.7)	34 (72.3)		5 (35.7)	9 (65.3)	
LDH			0.226			0.587
High	24 (30.4)	55 (69.6)		7 (29.2)	17 (70.8)	
Normal	32 (39.5)	49 (60.5)		15 (35.7)	27 (65.3)	
NLR, median (range)	2.59 (1.72–3.14)	2.87 (2.01–3.99)	0.033	2.80 (1.88–3.58)	2.9 (1.56-.3.90)	0.935
PLR, median (range)	128 (100.20–160.47)	139.74 (103.63–181.40)	0.158	127.37 (95.64–192.44)	148.26 (94.90–197.13)	0.924
Rad-score, mean ± SD	− 13.39 ± 3.39	− 17.58 ± 3.58	< 0.001	− 13.87 ± 3.30	− 16.81 ± 3.51	< 0.001

**Table 5:** Multivariate analysis of factors associated with CR status for ESCC patients received CCRT

Variables	Training set (n = 160)		Validation set (n = 66)	
	OR (95% CI)	*p*	OR (95% CI)	*p*
Clinical staging	0.260 (0.117–0.576)	0.001	0.392 (0.164–0.938)	0.035
Rad-score	1.355 (95%CI:1.180–1.556)	< 0.001	1.236 (1.029–1.484)	0.023

**Fig. 3:**
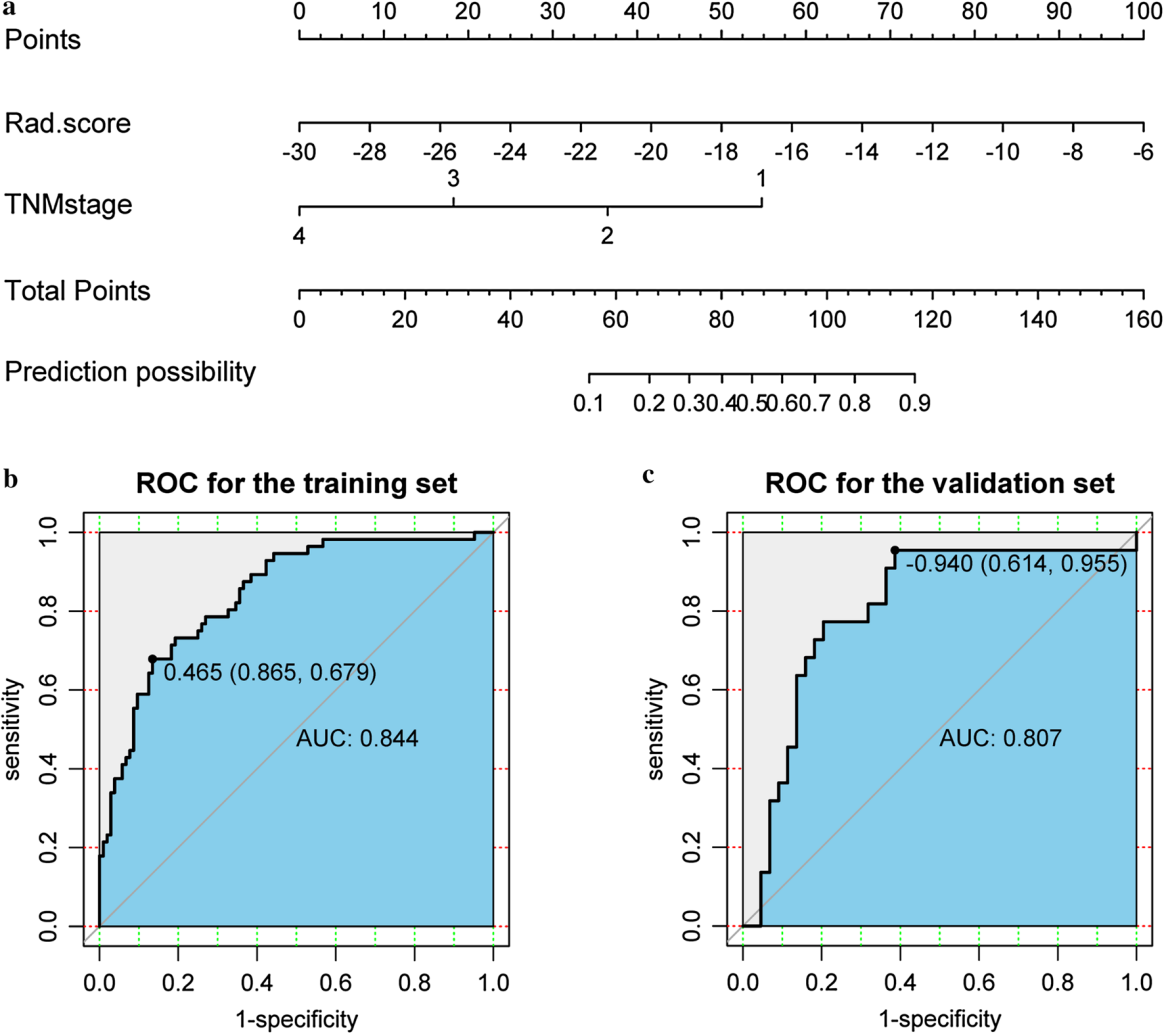
Development and validation of a predictive nomogram model for predicting CR status. **a** A predictive nomogram model combined Rad-score and clinical stage. **b**, **c** ROC curve for predictive model in training set and validation set

Based on the results of multivariate analysis, we developed a nomogram model as easy-to-use tool (Fig. 3a). As shown in Fig. 3b, c, the nomogram model performed well in CR status prediction for ESCC patients treated with CCRT in the training set with an AUC of 0.844 (95% CI 0.779–0.897), and showed similar discrimination in validation set (AUC = 0.807, 95% CI 0.691–0.894).

### Performance comparison of clinical staging and nomogram model

**Fig. 4:**
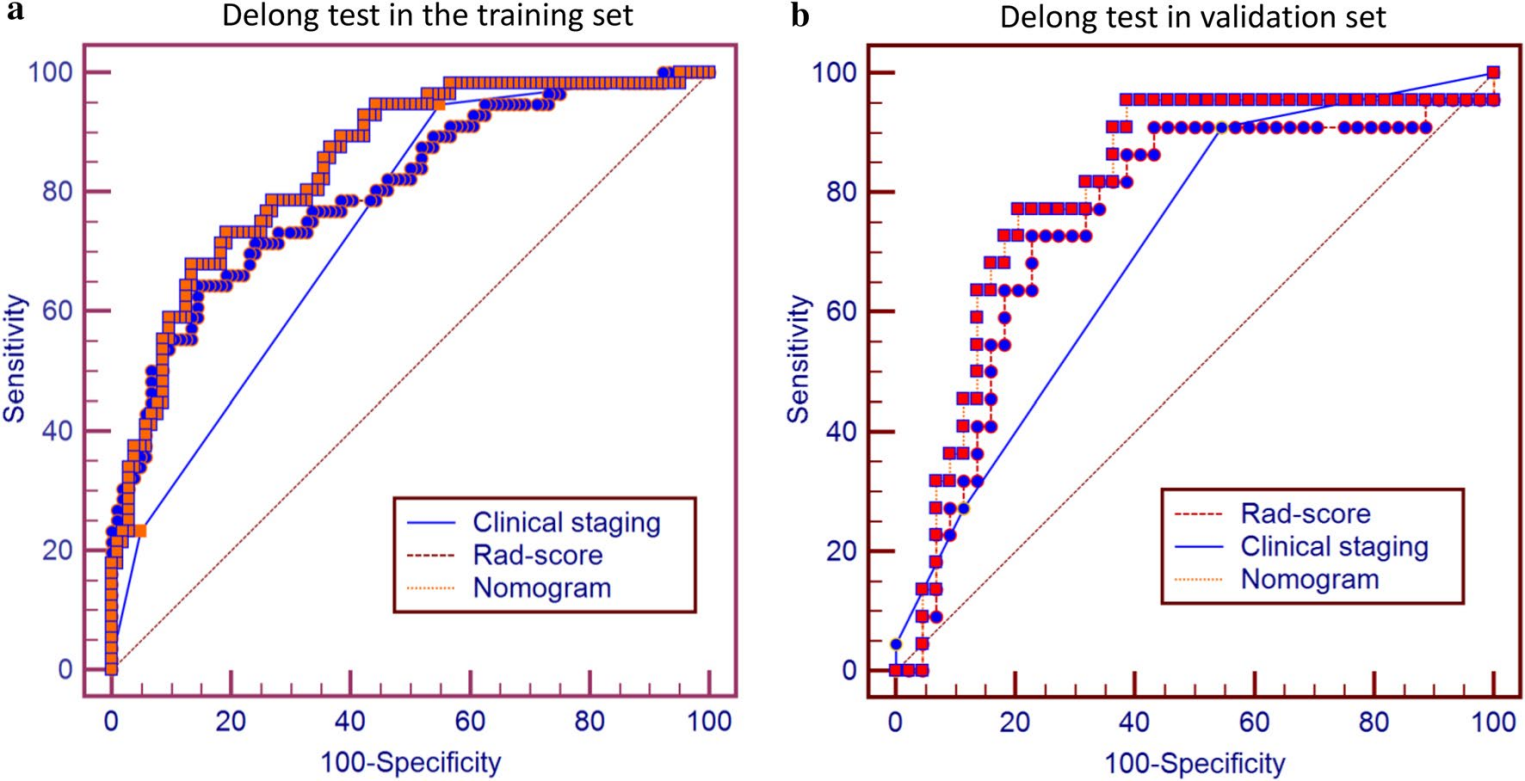
ROC curve comparison of nomogram model and clinical stage in training set (**a**) and validation set (**b**)

We further used the Delong test to compare the predictive performance of the nomogram model, Rad-score and the clinical staging for CR status. As shown in Fig. 4, the AUC of nomogram model was higher than that of the clinical staging, indicating that the nomogram model achieved considerably better discrimination capability than clinical staging both in the training set (DeLong's test, *p* < 0.001) and in the validation set (*p* = 0.026). The same result was found between the nomogram model and the Rad-score both in the training set (DeLong's test, *p* = 0.030) and in the validation set (*p* = 0.045).However, the difference between the Rad-score and the clinical staging was not significant both in the training set (*p* = 0.139) and in the validation set (*p* = 0.553).

### Clinical usefulness of the nomogram model

**Fig. 5:**
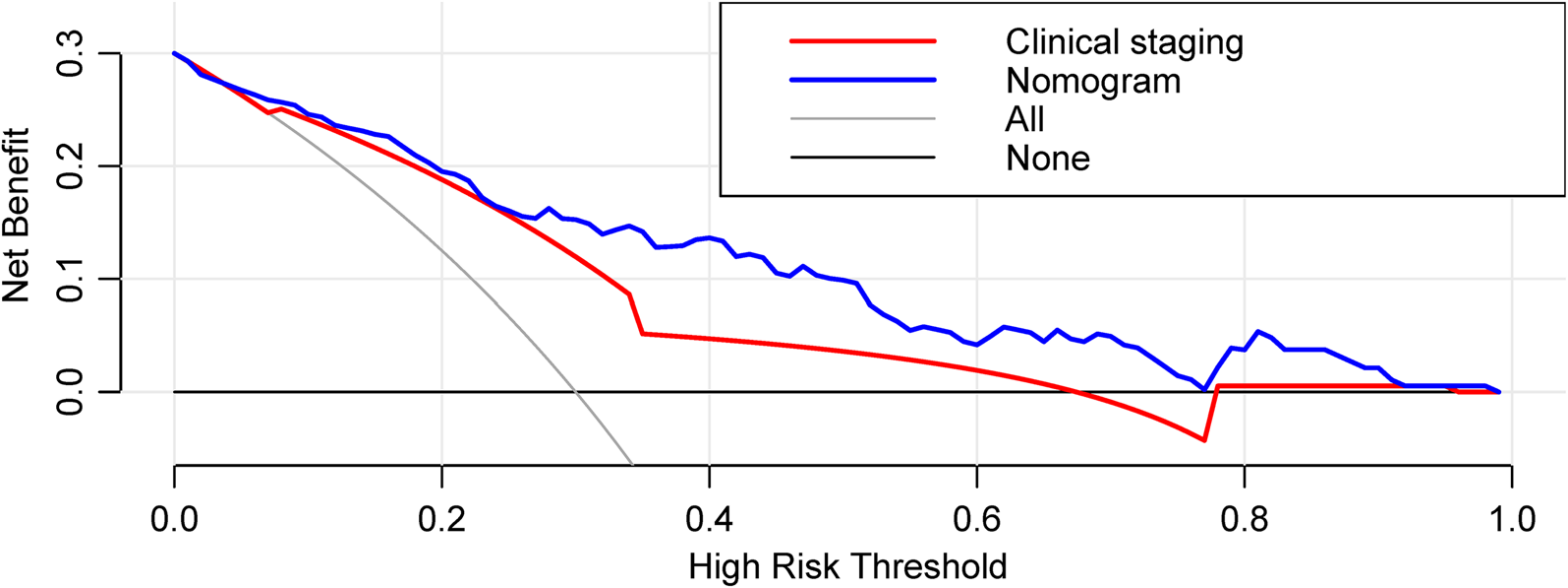
Decision curve analysis of the nomogram model

We used a decision curve to analyze the impact of the nomogram model on clinical treatment decisions. Through the decision curve, we can see that when the risk threshold was greater than 10%, the clinical staging or nomogram model was better than "all treatment" or "no treatment". When the threshold was greater than 25%, the predictive ability of the nomogram model was superior to clinical staging (Fig. 5).

## Discussion

For patients with ESCC who cannot be operated or refused surgery, CCRT was the main treatment. Previous studies have shown that patients who achieved CR after CCRT had a better prognosis than those didn’t achieve CR [7]. A predictive model of CR status would help us to classify patients' radio-sensitivity, and formulate more personalized treatment plans before treatment. In the present study, we developed and validated a nomogram model combined clinical staging and radiomics signature Rad-score for predicting CR status of ESCC patients treated with CCRT. The AUCs of the nomogram were 0.844 and 0.807 in the training set and the validation set, respectively, indicating a high predictive ability.

Radiomics is an emerging image analysis method, which can convert CT, MRI and PET-CT images into high-throughput radiomics feature data [12]. Investigators can extract radiomics features data from regions of interest by using the python software, including intensity, texture, shape, wavelet features, and so on. Then a radiomics signature was developed by linear or nonlinear machine learning methods to achieve a comprehensive quantitative description of the tumor for diagnosis, efficacy prediction and survival prognosis analysis [18]. Due to the advantages of radiomics feature signature over traditional imaging techniques, the application of radiomics to predict treatment response and prognosis has been launched widely. For patients with esophageal cancer who undergo concurrent chemoradiation, the treatment response assessment and prognosis prediction rely on medical image evaluation. However, there were many uncertainties, which had attracted attention of many investigators.

To date, several studies have reported the application of radiomics features in predicting the treatment response and prognosis for patients with esophageal cancer [10, 13, 14, 19–22]. Hou et al. [22] extracted 214 radiomics features from the pretreatment enhanced CT images of 49 patients with esophageal cancer. A model based on 5 radiomics features was developed with an AUC of 0.686–0.727 and the classification accuracy is 0.891 and 0.972, respectively. Li et al. analyzed the changes of CT radiomics features during the radiotherapy of esophageal cancer patients, and found that the tumor volume and CT value varied with the irradiation dose. Therefore, the CT based radiomics features can be used to early predict the response of chemoradiotherapy in patients with esophageal cancer. Jin et al. [14] combined CT radiomics features and dosimetric parameters to establish a model for predicting the response of esophageal cancer to chemoradiotherapy. However, the sample size included in previous studies was small. In our present study, 226 ESCC patients treated with CCRT were included for investigation. As a result, 7 CT radiomics features and clinical staging were selected to develop a nomogram model for predicting CR status of patients after CCRT. This model can provide an economic and non-invasive method for clinicians to predict the treatment response of ESCC patients treated with CCRT.

As we all know, tumor staging is the most important prognostic predictor for patients with malignant tumors, which is the basis for clinicians to make a treatment strategy. Previous study have found that patients with AJCC stage II were more likely to achieve CR after chemo-radiotherapy [23]. Other studies have shown that patients with more advanced T staging before treatment have a lower probability of achieving CR after CCRT [24]. In the T staging of esophageal cancer AJCC staging, the staging criteria are determined based on the depth of esophageal tumor infiltrating the esophagus wall and the relationship with the surrounding tissues and organs, which only represent the depth of the invasion of the esophageal cancer lesion in the horizontal axis direction but not the infiltration of the esophageal cancer lesion in the direction of the longitudinal axis of the esophagus [25]. Therefore, the primary tumor cannot be comprehensively evaluated due to deficiency of some prognostic information. In addition, the clinical staging of esophageal cancer relies on imaging examination, which was inevitably inconsistent with pathological staging. Radiomics analysis can extract the three-dimensional image information of tumors and provide information that comprehensively represents the tumor, consequently improving the accuracy of clinical staging. Our results showed that there was a significant correlation between the pretreatment clinical stage and treatment response of CR, with *p* < 0.001 in the training set and *p* = 0.016 in the validation set. Multivariate analysis showed that pretreatment clinical staging and radiomics signature Rad-score were independent predictors for CR status. Then a nomogram was developed and validated with AUCs of 0.844 in training set and 0.807 in validation set, respectively. Furthermore, we compared the performance of nomogram model and clinical staging and found that the prediction ability of the nomogram model was significantly better than clinical staging both in the training set and the validation set. These results suggested that the nomogram model we developed was superior to clinical staging in predicting chemoradiotherapy response.

Compared with previous studies, this study has its own advantages. First, the CT images collected in this study were the radiotherapy simulation positioning CT image, which were from the same CT machine with the unified scanning parameters to avoid the impact of different machines and different scanning parameters on radiomics features. Second, all esophageal cancer patients included in this study were pathologically diagnosed as esophageal squamous cell carcinoma and the sample size was the largest of its kind so far. Third, all patients received concurrent chemo-radiotherapy with a definitive radiation dose of 50–72 Gy.

Of course, this study also has several limitations. First of all, this study is a retrospective and single center investigation. The conclusion of the study still needs to be verified externally with larger sample size of patients. If possible, a prospective investigation will be more illustrative. Secondly, this study included only a few clinical features, and there were some confounding variables in this study, such as T stage T stage was correlated with clinical stage and the response to chemoraditoherapy, as well as correlated with the radiomics features which were extracted from the primary tumor lesion.Third, the treatment response evaluation was performed using CT images and barium esophagogram but not pathologically evaluation.

## Conclusion

In summary, we retrospectively analyzed the radiomics features of pretreatment CT images in ESCC patients treated with CCRT and developed a non-invasive, comprehensive, and personalized radiotherapy response prediction model. The nomogram model was combined radiomics signature Rad-score and clinical staging. This model provided us with an economical and simple method for evaluating the response of chemoradiotherapy for patients with ESCC.

## Supplementary information

**Additional file 1**. The list of radiomics features extracted from the delineated VOIs.

## Data Availability

The datasets used and/or analyzed during the current study are available from the corresponding author upon reasonable request.

## Abbreviations

ESCC: Esophageal squamous cell cancer
CCRT: Concurrent chemo-radiotherapy
CR: Complete response
LASSO: Least absolute shrinkage and selection operator
ROC: Receiver operating characteristic
AUC: Area under the receiver
EC: Esophageal cancer
CT: Computed tomography
MR: Magnetic resonance
3DRT: Three-dimensional conformal radiation therapy
GTV: Gross tumor volume
GTVnd: Nodal gross tumor volume
CTV: Clinical target volume
CTVt: Tumor clinical target volume
CTVnd: Nodal clinical target volume
PTV: Planning target volume
VOI: Volume of interest
GLCM: Gray-level co-occurrence matrix
GLSZM: Gray-level size-zone matrix
GLRLM: Gray-level run-length matrix
NGTDM: Neighboring gray-tone difference matrix
GLDM: Gray-level dependence matrix
